# A clinical tool to predict overall survival of elderly patients with soft tissue sarcoma after surgical resection

**DOI:** 10.1038/s41598-024-65657-2

**Published:** 2024-07-02

**Authors:** Xianmei Lv, Lujian Zhu, Gaochen Lan, Zhangheng Huang, Qiusheng Guo

**Affiliations:** 1 Department of Radiotherapy, Jinhua People’s Hospital, Jinhua, Zhejiang China; 2 Department of Infectious Diseases, Affiliated Jinhua Hospital, Zhejiang University School of Medicine, Jinhua, Zhejiang China; 3 Department of Oncology, The Second Affiliated Hospital of Fujian Medical University, Quanzhou, Fujian China; 4 Department of Orthopedics, West China Hospital, Sichuan University, Chengdu, Sichuan China; 5 Department of Medical Oncology, Affiliated Jinhua Hospital, Zhejiang University School of Medicine, No. 365 Renmin East Road, Jinhua, Zhejiang China

**Keywords:** Soft tissue sarcoma, Elderly, Nomogram, SEER database, Postoperative overall survival, Cancer prevention, Sarcoma, Risk factors

## Abstract

With the aging world population, the incidence of soft tissue sarcoma (STS) in the elderly gradually increases and the prognosis is poor. The primary goal of this research was to analyze the relevant risk factors affecting the postoperative overall survival in elderly STS patients and to provide some guidance and assistance in clinical treatment. The study included 2,353 elderly STS patients from the Surveillance, Epidemiology, and End Results database. To find independent predictive variables, we employed the Cox proportional risk regression model. R software was used to develop and validate the nomogram model to predict postoperative overall survival. The performance and practical value of the nomogram were evaluated using calibration curves, the area under the curve, and decision curve analysis. Age, tumor primary site, disease stage, tumor size, tumor grade, N stage, and marital status, are the risk variables of postoperative overall survival, and the prognostic model was constructed on this basis. In the two sets, both calibration curves and receiver operating characteristic curves showed that the nomogram had high predictive accuracy and discriminative power, while decision curve analysis demonstrated that the model had good clinical usefulness. A predictive nomogram was designed and tested to evaluate postoperative overall survival in elderly STS patients. The nomogram allows clinical practitioners to more accurately evaluate the prognosis of individual patients, facilitates the progress of individualized treatment, and provides clinical guidance.

## Introduction

Soft tissue sarcoma (STS) is a rare mesenchymal-derived tumor that most often occurs in the extremities. It is subdivided into about 75 distinct subgroups with distinct biology, molecular abnormalities, and therapy responses. Due to the rarity of such cancers and their numerous varieties, no large-scale data exist to guide treatment, necessitating a multidisciplinary and customized approach^1,2^. The incidence of STS in senior individuals has been growing in recent years, as the world ages^3,4^. Recent research indicates that STS instances occur in people 65 years of age, with the frequency of sarcoma increasing with age, and there is no conclusive idea regarding the best complete treatment for senior STS patients^5,6^. The current studies overall, these studies suggest that surgery in combination with radiotherapy remains the best means of controlling STS today compared to other treatments. Although increasing age shows a negative correlation with STS-specific survival, the prognosis for elderly patients with STS is favorable when older patients of appropriate age are selected to undergo extensive STS resection^7–9^. According to a study monitoring the epidemiology of STS, the analysis showed that fewer patients ≥ 85.5 years of age underwent surgical treatment^10^. Furthermore, recent studies suggest that STS cases gradually rise in patients 65 years and older. Therefore, it is important to study the clinical-related prognostic factors in STS elderly groups.

Given the relatively rare incidence of STS, clinical studies for STS are typically difficult, necessitating a more rigorous design that includes patient screening and therapy assignment. The nomogram combines and illustrates several important prognostic factors, is a reliable and valid tool for quantifying individual risk, and performs well in predicting survival in various cancers. In previous studies, nomograms for STS in specific tumor primary locations or STS of specific histological types (e.g. liposarcoma, fibrosarcoma) have been developed^11,12^. Callegaro et al. in 2016 reported an example of the nomogram for STS of the extremities and trunk, and the researchers also created specific nomograms for different STS with histological subtypes, tumor size, and tumor grade^13,14^. However, according to the current study, there are no reports on nomograms that predict overall survival (OS) after resection of primary tumors in elderly patients with STS. The goal of this study was to develop a nomogram for predicting postoperative OS in elderly STS patients using complete clinical data from the Surveillance, Epidemiology, and End Results (SEER) database. As a result, it's critical to gain a better knowledge of the clinicopathological and therapeutic distinctions in this population.

## Methods

### Study population and data collection

The data for this study was obtained from SEER*Stat Software. Medical ethical assessment is not required for the analysis of anonymous data in the SEER database, and informed permission is not required. Inclusion criteria: (1) the histological type of the patient is STS; (2) patient's age at diagnosis ≥ 65 years; (3) patients who have undergone resection of the primary tumor; (4) information on the patient's tumor characteristics (TNM stage, histological type, tumor size, and grade) and treatment (chemotherapy, radiotherapy) is complete. Exclusion criteria were as follows: (1) patients' survival time was less than one month; (2) patients’ treatment information was missing; (3) tumor-related characteristics were missing. Finally, we studied the postoperative prognostic factors in 2353 elderly patients with STS. In addition, these patients were diagnosed with STS between 2010–2015. The training set was for building the nomogram as well as the internal validation model, while the validation set was for external validation of the model.

Factors that may be related to the postoperative outcome of older STS patients were extracted, including age, sex, race, primary site, histological type, grade, T stage, N stage, M stage, tumor size, treatment information (radiotherapy, chemotherapy), disease stage, and marital status. The X-tile software determines the optimal cutoff of age for OS. Radiotherapy can be divided into no radiotherapy, pre-operative radiotherapy, and postoperative radiotherapy. The best cut-off values for age are 73 and 82 years, with the consequent division into three subgroups of 65–72, 73–82, and > 82 years. The primary site of the tumor is classified as the trunk, extremity, head, and neck.

### Statistical analysis

The total cohort was randomly assigned into a training set and a validation set in a ratio of 7:3. In the training set, univariate and multivariate Cox regression analyses were used to determine risk variables for the postoperative OS. Using R software, a model for the postoperative OS of patients was established based on the results of multivariate Cox regression analysis. In addition, receiver operating characteristic curves (ROC) were plotted and the area under the curve (AUC) was calculated to assess the discriminatory power. Development of calibration curves and decision curve analysis (DCA) for 1, 3, and 5 years to assess the predictive accuracy and clinical utility. Due to the limitations of the SEER database itself, the time of diagnosis is only accurate to the year, while it is difficult for us to obtain the follow-up time of the patients. R software (version 4.0.3, https://www.r-project.org/) was used for statistical analysis. The difference is statistically significant if the P-value is less than 0.05.

### Ethics approval and consent to participate

All methods were carried out by relevant guidelines and regulations. Data extraction and usage have been approved by the SEER Program. All the data can be found in the SEER dataset: https://seer.cancer.gov/seerstat/.

## Results

### Clinicopathological characteristics

All elderly STS patients who underwent resection of primary tumors surgery were randomized into two sets. In the training set, the vast majority were patients aged 65–72 years (43.18%) and 84.54% were white. Patients were predominantly graded IV (40.75%), T2 (67.50%), N0 (97.76%), and M0 (94.18%). Regarding treatment, 49.24% of patients did not undergo radiotherapy, 38.45% underwent postoperative radiotherapy, but most patients did not receive chemotherapy (89.81%), and most of the enrolled postoperative elderly patients with STS had predominantly localized tumors (74.65%). The histological type of tumors was predominantly liposarcoma (23.77%) and the size of tumors was predominantly > 100 mm (36.81%). The demographic and clinicopathological characteristics of postoperative elderly patients with STS were presented in Table 1.

**Table 1:** Clinical and pathological characteristics of elderly postoperative patients with STS.

Variables	Total cohort		Training cohort		Validation cohort	
	N = 2353		N = 1649		N = 704	
	n	%	n	%	n	%
Age						
65–72	1024	43.52	712	43.18	312	44.32
73–82	885	37.61	620	37.60	265	37.64
> 82	444	18.87	317	19.22	127	18.04
Race						
Black	159	6.76	108	6.55	51	7.24
Other	204	8.67	147	8.91	57	8.10
White	1990	84.57	1394	84.54	596	84.66
Sex						
Female	1020	43.35	710	43.06	310	44.03
Male	1333	56.65	939	56.94	394	55.97
Primary site						
Head and neck	161	6.84	114	6.91	47	6.68
Trunk	741	31.49	516	31.29	225	31.96
Extremity	1451	61.67	1019	61.80	432	61.36
Grade						
I	412	17.51	285	17.28	127	18.04
II	402	17.08	271	16.44	131	18.61
III	593	25.20	421	25.53	172	24.43
IV	946	40.20	672	40.75	274	38.92
T stage						
T1	753	32.00	536	32.50	217	30.82
T2	1600	68.00	1113	67.50	487	69.18
N stage						
N0	2299	97.70	1612	97.76	687	97.59
N1	54	2.30	37	2.24	17	2.41
M stage						
M0	2218	94.26	1553	94.18	665	94.46
M1	135	5.74	96	5.82	39	5.54
Radiotherapy						
No	1164	49.47	812	49.24	352	50.00
Radiation prior to surgery	301	12.79	203	12.31	98	13.92
Radiation after surgery	888	37.74	634	38.45	254	36.08
Chemotherapy						
No	2112	89.76	1481	89.81	631	89.63
Yes	241	10.24	168	10.19	73	10.37
Disease stage						
Localized	1744	74.12	1231	74.65	513	72.87
Regional	470	19.97	319	19.35	151	21.45
Distant	139	5.91	99	6.00	40	5.68
Tumor size						
< 50	687	29.20	483	29.29	204	28.98
50–100	790	33.57	559	33.90	231	32.81
> 100	876	37.23	607	36.81	269	38.21
Histological type						
Fibrosarcoma	44	1.87	27	1.64	17	2.41
Leiomyosarcoma	334	14.19	236	14.31	98	13.92
Liposarcoma	580	24.65	392	23.77	188	26.70
Malignant fibrous histiocytoma	227	9.65	158	9.58	69	9.80
Rhabdomyosarcoma	26	1.10	17	1.03	9	1.28
Synovial sarcoma	31	1.32	24	1.46	7	0.99
Other	1111	47.22	795	48.21	316	44.89
Marital status						
Unmarried	912	38.76	650	39.42	262	37.22
Married	1441	61.24	999	60.58	442	62.78

### Analysis of prognostic factors

First, univariate Cox regression analysis was performed on all variables in turn, showing that age, tumor grade, T stage, N stage, M stage, chemotherapy, primary site, disease stage, tumor size, and marital status were risk variables in postoperative elderly patients with STS. To exclude interactions between confounding factors, a multivariate Cox regression analysis was applied to these relevant predictive factors. Finally, independent predictive variables were found as age, grade, N stage, tumor size, primary site, disease stage, and marital status (Table 2).

**Table 2:** Analysis of univariate and multivariate Cox regression in elderly patients with soft-tissue sarcoma after surgery.

Characteristics	Univariate analysis		Multivariate analysis	
	HR (95% CI)	P value	HR (95% CI)	P value
Age				
65–72	Reference		Reference	
72–82	1.435 (1.187–1.734)	< 0.001	1.381(1.141–1.673)	0.001
> 82	2.574 (2.097–3.159)	< 0.001	2.382(1.922–2.952)	< 0.001
Race				
Black	Reference			
Other	0.780 (0.517–1.178)	0.238		
White	0.878(0.641–1.203)	0.419		
Sex				
Female	Reference			
Male	1.156(0.983–1.359)	0.080		
Histological type				
Fibrosarcoma	Reference			
Leiomyosarcoma	1.200 (0.623–2.309)	0.586		
Liposarcoma	0.772 (0.403–1.478)	0.435		
Malignant fibrous histiocytoma	1.446 (0.747–2.801)	0.274		
Rhabdomyosarcoma	2.055 (0.835–5.059)	0.117		
Synovial sarcoma	1.677 (0.712–3.951)	0.237		
Other	1.444 (0.769–2.710)	0.253		
Grade				
I	Reference		Reference	
II	2.169 (1.508–3.120)	< 0.001	2.453 (1.695–3.551)	< 0.001
III	3.999 (2.900–5.515)	< 0.001	3.845 (2.769–5.340)	< 0.001
IV	3.563 (2.608–4.867)	< 0.001	3.500 (2.542–4.819)	< 0.001
T stage				
T1	Reference			
T2	1.546 (1.288–1.856)	< 0.001		
N stage				
N0	Reference		Reference	
N1	2.753 (1.844–4.110)	< 0.001	1.836(1.201–2.806)	0.005
M stage				
M0	Reference			
M1	3.967 (3.093–5.089)	< 0.001		
Radiotherapy				
No	Reference			
Radiation prior to surgery	1.103 (0.859–1.417)	0.441		
Radiation after surgery	1.884 (0.744–1.050)	0.161		
Chemotherapy				
No	Reference			
Yes	1.785 (1.426–2.236)	< 0.001		
Tumor size				
< 50	Reference		Reference	
50–100	1.392 (1.121–1.729)	0.003	1.485 (1.181–1.867)	0.001
> 100	1.744 (1.418–2.145)	< 0.001	2.134 (1.678–2.699)	< 0.001
Primary site				
Head and neck	Reference		Reference	
Trunk	0.796 (0.596–1.602)	0.121	0.601(0.439–0.824)	0.002
Extremity	0.563 (0.426–0.744)	< 0.001	0.493 (0.343–0.625)	< 0.001
Disease stage				
Localized	Reference			
Regional	1.473 (1.214–1.788)	< 0.001	1.165 (0.946–1.434)	0.150
Distant	4.246 (3.308–5.449)	< 0.001	3.156 (2.414–4.125)	< 0.001
Marital status				
Unmarried	Reference		Reference	
Married	0.694 (0.591–0.814)	< 0.001	0.808 (0.684–0.955)	0.012

### Prognostic nomogram

**Figure 1:**
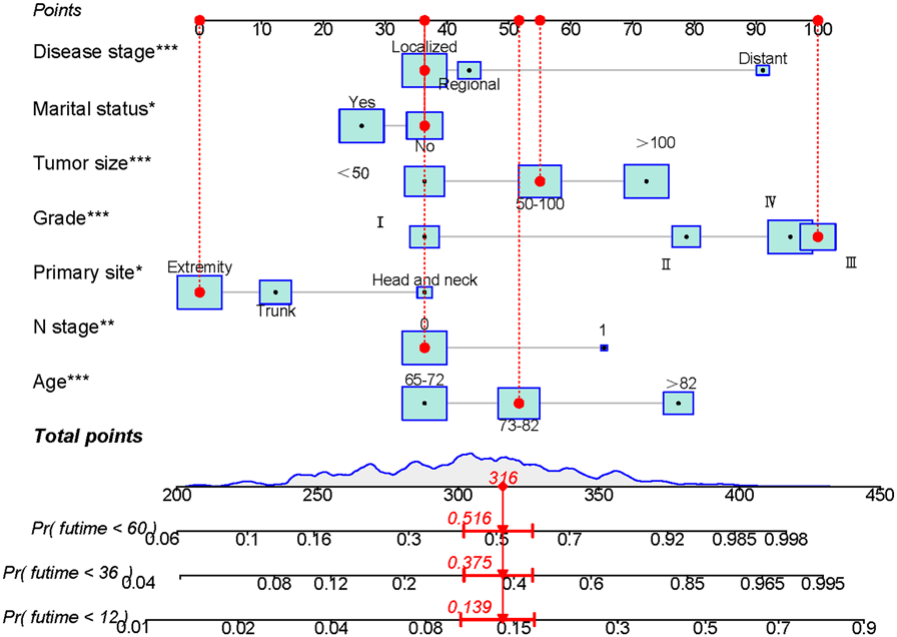
Nomogram for predicting 1, 3, and 5-year overall survival. An unmarried patient who has a localized tumor measuring 75 mm with a grade III tumor classification and a primary site in the right upper extremity. Then his predicted overall survival rates at 1, 3, and 5 years are 86.1, 62.5, and 48.4%, respectively.

A nomogram was created to predict the postoperative OS based on the screening of relevant predictive variables (Fig. 1). As shown in Fig. 1, the primary site of the extremity was a protective factor for elderly postoperative patients with STS, and the prognosis of elderly postoperative patients with STS of the extremities was better compared to that of STS of the trunk. Marital status had less impact on the prognosis of elderly postoperative patients with STS. Elderly postoperative patients with STS aged > 82 years had a worse prognosis compared with elderly postoperative patients of other ages. The OS of patients with tumor size > 100 mm was worse, and the OS of patients with STS who had distant metastases was worse than those who did not have metastases.

### Validation of the nomogram

**Figure 2:**
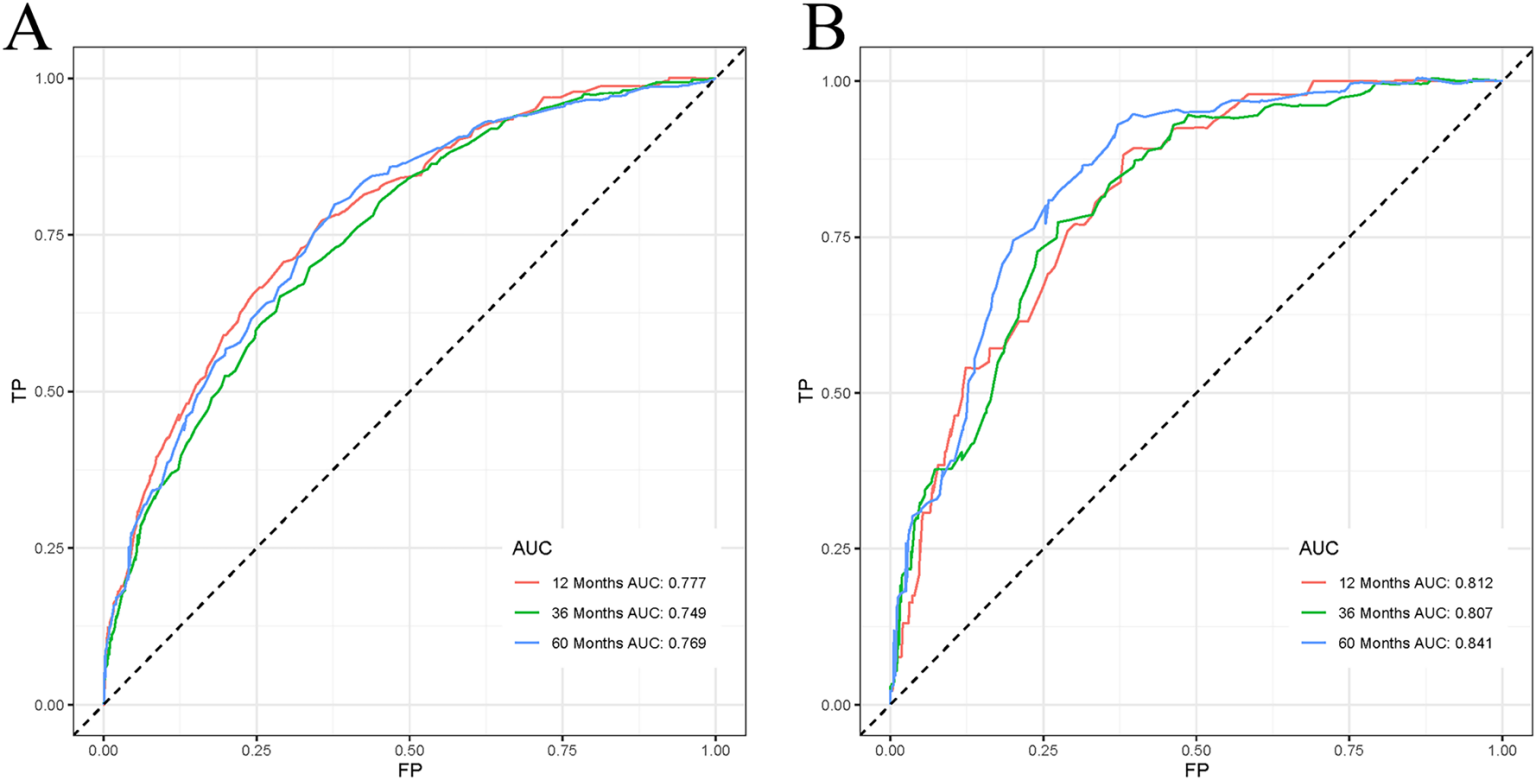
Receiver operating characteristic curves for elderly patients with soft tissue sarcoma after surgical resection. (**A**) Training set, (**B**) Validation set.

**Figure 3:**
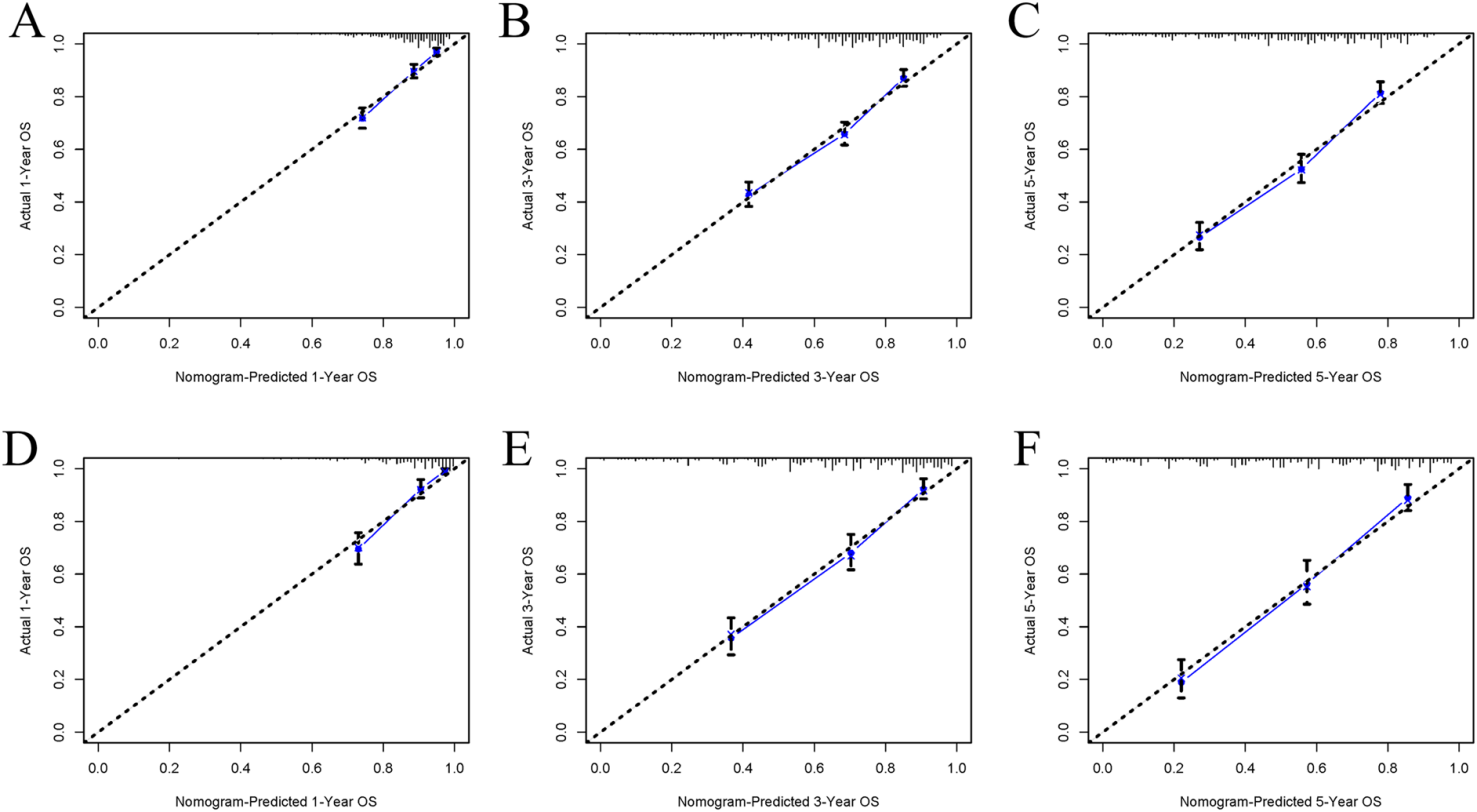
Calibration of the nomogram model in the training set (**A**–**C**), and validation set (**D**–**F**), respectively.

**Figure 4:**
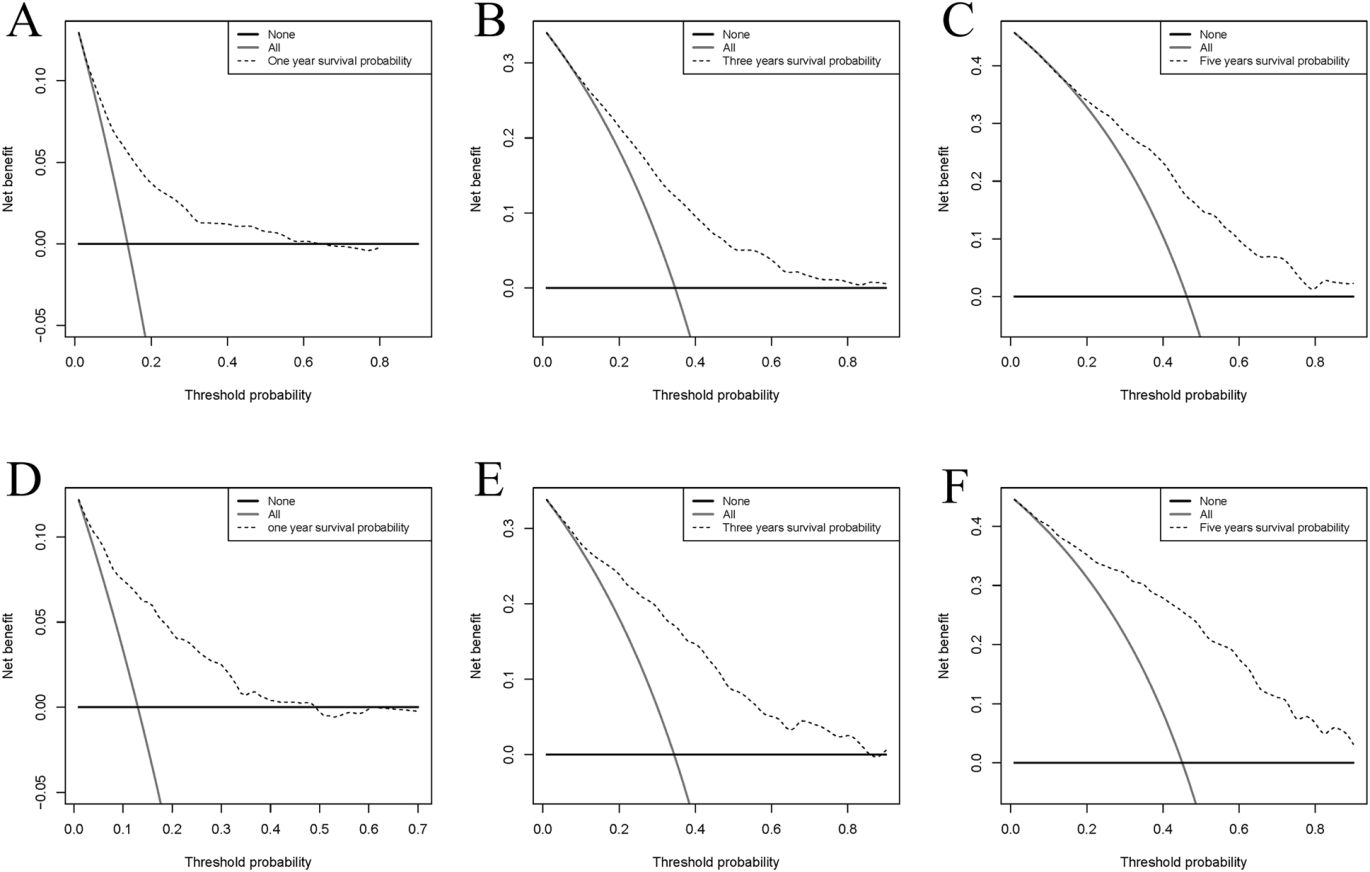
Decision curve analysis of the nomogram model in the training set (**A**–**C**), and validation set (**D**–**F**), respectively.

The AUC for 1, 3, and 5 years was 0.777, 0.749, and 0.769 in the training set and 0.812, 0.807, and 0.841 in the validation set, respectively, all of which demonstrated good discrimination for predicting between models (Fig. 2). In the calibration curve, the curve is close to the sloping 45° line, which indicates a good level of agreement between the nomogram’s projected and actual results (Fig. 3). In both the training and validation sets, DCA demonstrated the good clinical usefulness of the nomogram in predicting postoperative OS in elderly patients with STS, as shown in Fig. 4.

### Risk classification system

**Figure 5:**
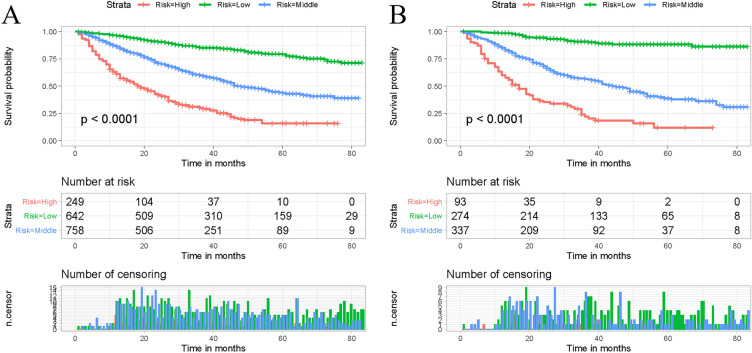
Kaplan–Meier survival analyses were performed for comparing postoperative overall survival in the low risk, middle risk, and high risk subgroups of all patients in the training set (**A**) and validation set (**B**).

The optimum cut-off point of the overall score based on OS was determined using X-tiles software, and all cases were classified into low risk (295), middle risk (295–340), and high risk (> 340) groups. Kaplan–Meier survival curves for each risk subgroup were plotted and log-rank tests were performed, which showed differences in OS among patients with different risk levels (P < 0.001) (Fig. 5). Patients in the high risk group had a worse outcome than those in the low risk group in the training and validation set. It suggests that the nomogram-based risk classification system seems to have a high predictive ability for the OS.

## Discussion

In this study, a clinical prediction model was constructed by the SEER database to predict postoperative OS in elderly patients with STS. The nomogram we created, as shown in Fig. 3, can accurately predict OS at 1, 3, and 5 years. This is the first study to use extensive and diverse case data to build a predictive nomogram model for elderly postoperative patients with STS. This prediction nomogram can easily predict patients’ prognosis, inform patients of the benefits of certain treatments, and have important implications for clinical decision-making.

It’s vital to keep in mind that not all groups of elderly adults with STS can be surgically treated. In the present results, the prognosis of elderly postoperative patients with STS > 82 years of age was worse compared to patients in the 73–82 years age group. It was similarly confirmed in previous single-center institutional studies that age is not a contraindication to surgery in elderly cancer patients with STS^15–17^. Buchner et al. prospectively studied 21 STS patients over the age of 70, and they discovered that even after surgery, these patients had a worse 5-year OS^18^. Lahat et al. analyzed 325 STS ≥ 65 years of age patients while selecting appropriate elderly patients among these individuals for surgery. They noted a decrease in bit survival after surgery in patients > 75 years of age. However, postoperative adverse effects and recovery time did not differ significantly between these STS age groups^7^. In another study it was also confirmed that surgery is safe and that reduced surgical use in the elderly may be an area of improved prognosis, while this study also noted a significant reduction in mortality in the elderly at 90 days after undergoing STS, suggesting that the postoperative 90-day period is key to the increased surgical risk affecting elderly patients with STS. Whether these factors influence surgical decision-making in elderly patients directly or indirectly^19^. Taken together, each of these studies concludes that despite the reduced OS in the elderly patient population after surgery compared to the younger patient population, surgery is still advisable for the relatively low perioperative complication rate and postoperative adverse effects in a subset of appropriate elderly patients with STS.

Whether tumors metastasize or not, disease stage and tumor size also have a different prognosis for elderly patients with STS after surgery. Elderly patients with STS who developed distant metastasis in this study had a poorer OS in comparison to elderly patients with localized tumors. The poorer prognosis after surgery in elderly patients with STS with tumor diameter size > 100 mm is an important disadvantage. Several studies to date have confirmed the prognostic factors associated with the diagnosis of STS in adults. In previous studies, the most common unfavorable prognostic factors for elderly postoperative patients with STS were found to be: (1) Site of tumor occurrence: including head, neck, and trunk, (2) different subtypes of tumors, (3) deeper tumor location, (4) residual tumor cells are still present at the surgical incision margin, (5) tumor size > 5 cm, and other factors^20–24^. Previous research has indicated that younger STS patients are more likely than older STS patients to develop tumors in a deeper position. Smaller tumor diameter and lower tumor grade were found to be independent risk variables for recurrence-free survival and OS in younger and older STS patients, even though older STS patients had larger tumors and a higher proportion of graded tumors^25^. Therefore, compared with younger patients with STS, older patients have relatively larger tumors, higher tumor stage, and grade, and are prone to distant metastases, causing some surgical difficulty and making surgical decisions difficult, which may also contribute to the poorer prognosis of older patients with STS.

## Conclusion

We constructed and verified a predictive nomogram to estimate the personalized postoperative OS of elderly patients with STS. The nomogram allows clinical practitioners to more accurately evaluate the prognosis of individual patients, facilitates the progress of individualized treatment, and provides clinical guidance.

## Data Availability

The dataset from the SEER database that was generated and/or analyzed during the current study is available in the SEER dataset repository (https://seer.cancer.gov/). The datasets generated during and/or analyzed during the current study are available from the corresponding author on reasonable request.

## Abbreviations

*STS*: Soft tissue sarcoma
*OS*: Overall survival
*SEER*: Surveillance, epidemiology, and end results
*ROC*: Receiver operating characteristic curves
*AUC*: Area under the curve
*DCA*: Decision curve analysis
